# The ‘stealth-bomber’ paradigm for deciphering the tumour response to carbon-ion irradiation

**DOI:** 10.1038/s41416-022-02117-6

**Published:** 2023-01-13

**Authors:** Anne-Sophie Wozny, Claire Rodriguez-Lafrasse

**Affiliations:** 1 Cellular and Molecular Radiobiology Laboratory, UMR CNRS5822/IP2I, Univ Lyon, Lyon 1 University, Lyon-Sud Medical School, Oullins, France; 2 Department of Biochemistry and Molecular Biology, Lyon-Sud Hospital, Hospices Civils de Lyon, Pierre-Bénite, France

**Keywords:** Radiotherapy, Cell biology, Radiotherapy

## Abstract

Numerous studies have demonstrated the higher biological efficacy of carbon-ion irradiation (C-ions) and their ballistic precision compared with photons. At the nanometre scale, the reactive oxygen species (ROS) produced by radiation and responsible for the indirect effects are differentially distributed according to the type of radiation. Photon irradiation induces a homogeneous ROS distribution, whereas ROS remain condensed in clusters in the C-ions tracks. Based on this linear energy transfer-dependent differential nanometric ROS distribution, we propose that the higher biological efficacy and specificities of the molecular response to C-ions rely on a ‘stealth-bomber’ effect. When biological targets are on the trajectories of the particles, the clustered radicals in the tracks are responsible for a ‘bomber’ effect. Furthermore, the low proportion of ROS outside the tracks is not able to trigger the cellular mechanisms of defence and proliferation. The ability of C-ions to deceive the cellular defence of the cancer cells is then categorised as a ‘stealth’ effect. This review aims to classify the biological arguments supporting the paradigm of the ‘stealth-bomber’ as responsible for the biological superiority of C-ions compared with photons. It also explains how and why C-ions will always be more efficient for treating patients with radioresistant cancers than conventional radiotherapy.

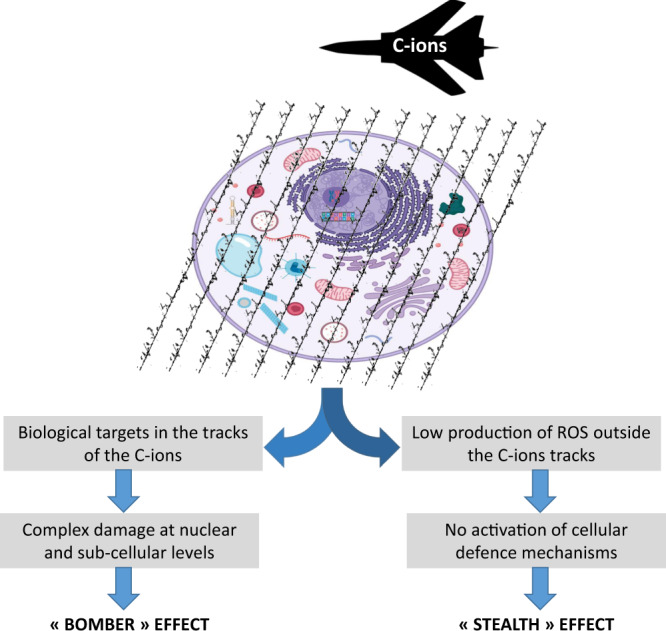

## Introduction

Carbon-ion irradiation (C-ions) presents physical and biological advantages compared with photons. These high-linear energy transfer (LET) particles deposit most of their energy at the end of their course, during the so-called Bragg peak, allowing them to target deep-seated tumours near critical anatomical structures and to spare normal tissues [1, 2]. Their greater radiobiological efficacy is demonstrated by an enhanced relative biological effect (RBE) and a decreased oxygen effect, in and around the Bragg peak area. This leads to less dependence on the cell cycle phase and enhanced cell killing, especially on hypoxic and cancer stem cells [1, 3–5]. This increased RBE relies partially on the higher deposition of energy in the biological matter (tissues, cells, organelles), which triggers complex clustered DNA lesions (single and double-strand breaks (DSBs), base damages) in the particle tracks that are very difficult to repair, and damage in chromatin structures such as chromosome aberrations [6–8]. Before running out of energy, particles create a fairly straight ionisation track, with electrons ejected along the track, compared with photons, which induce homogeneous and diffuse ionisation [2].

Indeed, radiation acts directly and indirectly on biological targets. The direct effects occur through direct interaction with the DNA molecules by breaking their bonds, then inducing a cascade of deleterious biological events [9, 10]. In contrast, the indirect effects relying on water radiolysis lead to a powerful induction of radical species such as reactive oxygen species (ROS), which trigger molecular alterations of signalling events detrimental to cell survival [10, 11]. High levels of ROS produce DNA damage, lipid peroxidation, modification of the membrane permeability disrupting homoeostasis, or oxidation of the amino acids associated with changes in the three-dimensional structure of proteins [12]. If the indirect effects are predominant in response to low-LET irradiation (photons) because water constitutes 60% of the biological tissues, the effects of high-LET exposure on biological structures result in enhanced direct and indirect effects [9, 13]. The dense clustering of ionisation in the track structure affects the biological targets at a cellular and molecular level through the localised production of ROS, which recombines with each other to form secondary molecular species. In fact, the major production of ROS relies on O_2_^•–^ and HO_2_^•^ radicals, which evolute with the partial pressure of oxygen and various LET to produce major species such as hydroxyl radicals (OH^•^), hydronium ions (H_3_O^+^) and solvated electrons (e_aq_^−^), mainly produced from the single ionisation of water molecules [14].

**Fig. 1:**
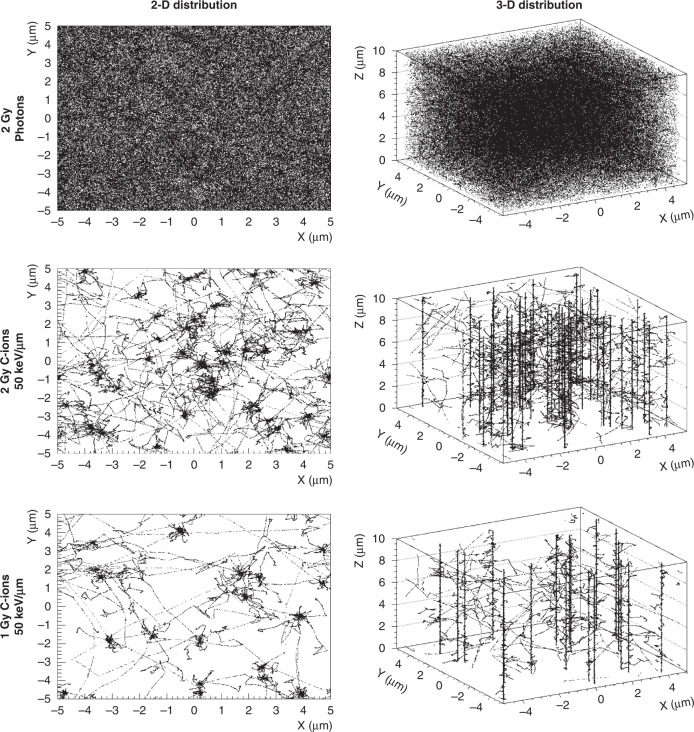
Monte Carlo simulations of the OH^•^ radical distribution in water in response to photons and to a mixed radiation field of C-ions (dose-averaged linear energy transfer (LET) ~50 keV/μm) at physical and biological equivalent doses of 2 Gy photons. Figures represent the superimposition of the radical distributions produced 10^−12^ s after the impact of each particle for doses of 2 Gy deposited by photons and 1 or 2 Gy by C-ions in the order of magnitude of the nucleus dimensions (10 × 10 × 10 μm). Simulations with photons are adapted from ref. [4] (https://www.mdpi.com/2072-6694/11/4/468/htm CC BY 4.0), and simulations with C-ions were reproduced with the permission of Dr Caterina Monini and Prof Michael Beuve.

In our previous work, Monte Carlo simulations at 13 keV/μm showed at 10^−12^s that the OH^•^ produced in a volume of 10 × 10 × 10 μm were 519,049 radicals in response to 2 Gy C-ions and 422,943 radicals in response to 2 Gy X-rays. Because OH^•^ are the most reactive radicals leading to cell killing, we hypothesised that it is their different spatial distribution at the nanometric scale rather than their relative concentration that could explain the higher biological efficacy of C-ions compared with photons. Furthermore, to confirm this different spatial distribution, Monte Carlo simulations of OH^•^ were performed at another LET of 50 keV/μm at physical and biological equivalent doses (Fig. 1).

**Fig. 2:**
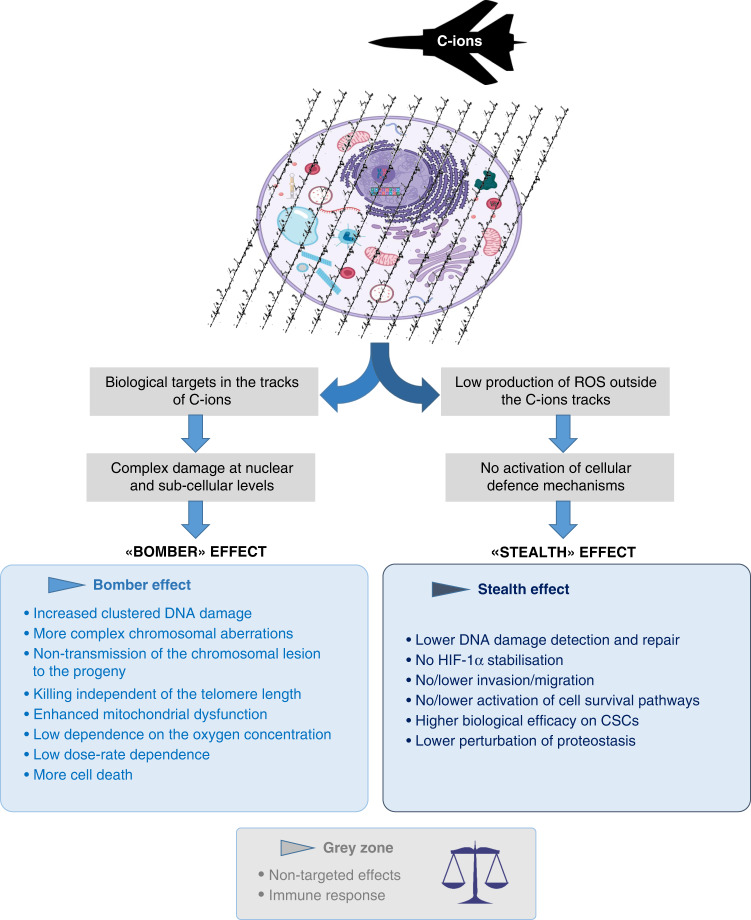
Diagram displaying the ‘stealth-bomber’ paradigm. Specific biological advantages of C-ions support, respectively, the ‘bomber’ and ‘stealth’ effects of C-ions.

Based on the differential distribution of radicals at a nanometric scale according to the LET, we propose that the higher biological efficacy of C-ions relies on a ‘stealth-bomber’ effect. On the one hand, the clustered radicals in the tracks of the particle would be responsible for a ‘bomber’ effect when biological targets such as DNA or organelles are on their trajectories. On the other hand, C-ions deceive cellular defences through the ‘stealth’ effect. Unlike X-rays, the absence of significant ROS production outside the C-ion tracks does not allow the achievement of a decisive ROS threshold necessary to activate the survival and defence pathways of the cancer cells (Fig. 2).

This review discusses which specific biological advantages of C-ions support the ‘bomber’ and ‘stealth’ effects of C-ions.

## Bomber effects on cancer cells

As mentioned above, ROS are produced in the tracks of the particles after C-ion exposure. When biological targets are on their trajectories, ROS induce complex and deleterious damage to the cancer cells. This condensed production of ROS at the nanometre scale contributes to explaining most of the deleterious properties of C-ions on cancer cells at molecular and cellular levels and could be categorised as a ‘bomber’ effect.

### At the nucleus level

#### Clustered DNA damage

The clearest evidence of the ‘bomber’ effect arises from the capacity of C-ions to produce complex and clustered lesions of the DNA also called locally multiply damaged sites (LMDS) [15, 16]. This clustered damage has been defined as two or more bistranded lesions (single-strand break, DSB, oxidised bases and apurinic-apyrimidinic (abasic) sites…) within 10–20 base pairs, which corresponds to 1–2 helical turns of the DNA molecule [7]. It is generally assumed that LMDS result from a direct interaction of the particles with the biological matter but also from the densely packed ionisation in the particle track structure [17]. In vitro experiments and Monte Carlo simulations showed that C-ion exposure induces a higher proportion of clustered DSBs than photons, therefore difficult to repair, probably because of a spatial crowding of the lesion site and/or reduced or inhibited enzymatic repair activities [18]. Because of their high complexity in terms of types of lesions, numbers of lesions per cluster, or their spatial distribution, each clustered lesion is different. High-resolution microscopy is required to describe these radiation-induced clusters [19, 20]. Moreover, the composition of the LMDS and the following repair pathways involved could depend on the cell type but also on the dose and the LET, which adds another level of complexity to their repair [20]. A recent study investigated the contribution of the direct effects of C-ions compared with their indirect action through the prism of OH^•^ formation in relation to the cell killing resulting from clustered damage [21]. They concluded that the efficacy of C-ions in cell death results from the contribution of OH^•^ produced in the track, which is in favour of the ‘bomber’ effect.

#### Chromosomal aberrations

##### Formation

After low- and high-LET irradiation, chromosomal breakages are the most prominent type of damage produced, which remain mostly unrejoined even after a long repair time following C-ion exposure. Moreover, the residual breaks often reflect the intrinsic radiosensitivity of normal and tumour cells [16, 22]. Many studies have investigated the effects of particles distributed along track structures on the induction of chromosomal aberrations at different LET, with different heavy particles and different models (normal or tumour cells, murine models) [23, 24]. By consensus, the induction of chromosome damage by C-ions arises during the first cell division at very high levels, especially for isochromatid breaks and complex rearrangements [25]. The higher efficacy of C-ions in inducing isochromatid breaks reflects the track structure of the heavy ions compared with dense and diffuse ionising radiation. Besides, fluorescence in situ hybridisation confirmed that heavy ions, even at low doses, increased the complexity of chromosome rearrangements, as well as the complex-type exchanges composed of at least 3 breaks in at least 2 chromosomes [26]. However, if interchanges between chromosomes are relatively well described and frequent after C-ions, the intra-changes seem to be less frequent at the first mitosis [27].

##### Transmission

Another interesting aspect of the complexity of the aberrations induced by C-ions is the non-transmission of the chromosomal lesions to the progeny of irradiated tumour cells, which would limit the genomic instability, thereby improving the local control [16]. By contrast, sparse ionising radiation induces the transmission of complex rearrangements to future cell generations [28]. Following C-ion exposure, the distribution of rearrangements and aberrations in each cell are highly overdispersed, leading to an RBE close to 1 in the progeny of irradiated cells and thus confirming the lack of transmission, but also the low probability of secondary cancers or local recurrences [28]. These observations could have a strong impact on the understanding of the potential later consequences of irradiation with heavy particles. Moreover, irradiation induces epigenetic modifications such as a decrease in the DNA methylation and acetylation of some histones but with different patterns. It was shown that although the epigenetic response is quite similar after low- and high-LET radiation, it depends more on the type of particles and the track structure than on the LET [29]. Altogether, these data highlight the ‘bomber’ effect of C-ions, relying on the complex chromosomal aberrations in the track structure.

#### Telomeres length

At the nuclear levels, the independence of the response to C-ions with respect to the telomeric status of tumour cells can also support the ‘bomber’ effect of C-ions. Low- and high-LET radiations produce major DNA damage and the same type of cell death. Ionising radiation and C-ion exposure are both expected to play the same role in telomere damage of the tumour cells. Surprisingly, we established that the efficacy of C-ions is independent of the telomere size [30]. Indeed, although long telomeres are a sign of resistance to photons in glioblastoma cell lines, the radiosensitivity to C-ions remains the same regardless of the telomeres’ size. By contrast, the dense and homogeneous formation of ROS in response to photons is particularly reactive on the guanine-rich (T2AG3 repeats) telomeric sequences, which are very sensitive to oxidative stress [31]. This makes short telomeres a preferential target within the genome after low-LET irradiation [32]. However, C-ions produce LMDS along the tracks of the particles. For this reason, even if some telomeres are on the path of the particles, they represent a small proportion of the genome and they should not be more damaged than the other sequences of the genome. All of these data highlight that patients with long telomeres, such as glioblastoma patients, can advantageously benefit from carbon therapy. Because of the physical properties of C-ions, the ‘bomber’ effect resulting from exposure to C-ions is telomere-length independent.

### At membrane levels

Ionising radiation can interact with the cellular membranes, leading to the production of ceramide, a fundamental mediator of apoptosis [33]. In head and neck squamous cell carcinoma (HNSCC) and glioblastoma cell lines, photon and C-ion exposure induced an early ceramide production in radiosensitive cells and a delayed one in radioresistant cells [34]. Unlike radiosensitive cells, which activate a time, dose and LET-dependent apoptosis through the ceramide pathway, radioresistant cells can activate a process of mitotic catastrophe ending in ceramide-dependent late apoptosis [34–36]. After C-ion exposure, no modification of the type of cell death occurred when compared with photons. Nevertheless, a prolonged cell cycle delay (G2/M arrest) is triggered, consistent with the complexity of the clustered lesions and their difficulty in being repaired [35]. Ceramide acts upstream of the mitochondrial collapse and the caspase activation, suggesting its key role in the ‘bomber’ effect when cellular membranes are on the path of the heavy ions [34].

Some reports suggested that the ceramide-dependent apoptosis pathway is independent of the *p53* status, although the lack of ceramide production has been associated with radioresistance [33, 34]. Considering that *p53* is mutated in more than 50% of the tumours [37], it is very interesting to note that apoptosis induced by C-ions would be independent of the *p53* status, making high-LET radiation efficient on a wide variety of radioresistant cells [35]. Altogether, these studies allowed us to conclude that ceramide is a determining factor and the molecular bridge between mitotic death and delayed apoptosis in response to high-LET radiation [34]. When cellular membranes are localised in the clustered ionising tracks, C-ions could overcome the resistance of cancer cells by a ‘bomber’ effect through the activation of the ceramide apoptotic pathway, independently of the *p53* status.

Radiation also induces lipid peroxidation, which causes ROS production, and amplifies the lipid peroxidation process leading to cell membrane damage, and, thus, cell damage such as the thickness of the membrane or holes. Ferroptosis, as a regulated cell death mechanism, results from the excessive production of lipid peroxidation and redox-active iron. This leads to shrunken mitochondria, increased membrane density, and rupture of the outer mitochondrial membrane. There are more and more reports, that suggest the activation of this mechanism by radiation [38].

### At the mitochondria level

**Fig. 3:**
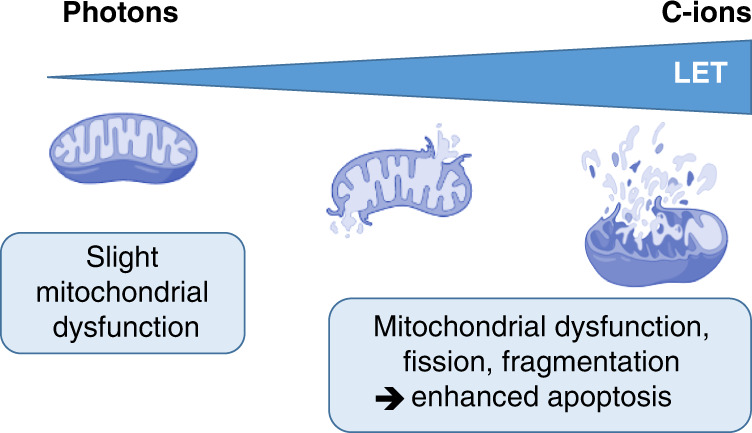
‘Bomber’ effect of C-ions at the mitochondrial level. As the LET increases, the damage to mitochondria is enhanced, such as dysfunction, fragmentation, or fission, leading to apoptosis.

Although researchers in the radiation biology field have extensively worked on the DNA-damaging effects of irradiation, the consequences of mitochondria exposure to low-LET and high-LET radiations have been more recently investigated. Mitochondria play a central role in cell metabolism by providing energy, in the form of adenosine triphosphate (ATP) via the oxidative phosphorylation metabolic pathway necessary for maintaining cellular integrity, cell survival and homoeostasis [39]. They are the principal source of physiological ROS (O_2_^–•^), which can be enhanced by irradiation and then leak out of the mitochondria to induce cytoplasmic and nuclear damage [29]. The ROS generated by irradiation can then induce programmed cell death (apoptosis). Because cancer cells present high metabolic and proliferative activities associated with increased mitochondria mass, the mitochondria of cancer cells are more vulnerable to irradiation [40]. Usually, photons induce slight mitochondrial dysfunction, fission and fusion, whereas C-ions produce enhanced mitochondrial dysfunction, fusion, lesions and fragmentation of mitochondrial DNA associated with increased apoptosis [41] (Fig. 3). Compared with photons, this high capacity of C-ions to induce apoptosis prevents cancer cells from escaping programmed cell death and is associated with reduced autophagy and mitophagy, contributing to the higher efficacy of C-ions. Indeed, the initial track concentration of O_2_ is estimated to be three times higher than the oxygen levels present in normally oxygenated and hypoxic tumour regions [42]. Concomitantly, the anti-oxidant enzymes decrease after C-ions [43]. Moreover, compared with photons, C-ions increase Bax levels and decrease the expression levels of anti-apoptotic protein Bcl-2. Here, compared with low-LET radiation, the high density of ROS in the tracks of C-ions strongly affects mitochondria, particularly in cancer cells, by inducing enhanced fragmentation, fission, the release of mitochondrial DNA and initiation of apoptosis, supporting the ‘bomber’ effect of C-ions.

### Cellular response to exogenous factors

#### Oxygen concentration

In solid tumours, hypoxia promotes radioresistance to conventional radiotherapy, particularly in acute conditions under 0.4–4% of oxygen [44]. Much evidence supports the weak dependence of C-ions on oxygen concentration. At cellular levels, the oxygen enhancement ratio (OER) is defined as the ratio of doses required to induce the same biological effect (usually 10% cell survival) under hypoxic and oxic conditions [45]. In response to photons, the OER can be about 3, as well as with low-LET particles. However, by increasing the LET, the OER decreases, associated with a lower dependency of the cells on the oxygen tension and, therefore, a higher sensitivity to radiation, especially with C-ions [46]. A nice study highlighted a decrease of the OER, from 3 with photons, to below 2 at 100 keV/μm, and to reach a minimum above 300 keV/μm in cancer cell lines irradiated with particles [47]. The LET-dependence of OER was confirmed in a wide variety of cancer cell lines but poorly influenced by the dose applied and without any significant difference between acute and chronic hypoxia [3, 48–50]. Preclinical studies, although less numerous, reported a decrease of the OER along the Spread-Out Bragg Peak (SOBP) of C-ions compared with photons in tumours [51]. In patients with uterine cervical cancer and treated with C-ions, the differences in partial pressures of intratumoral oxygen (normoxic and hypoxic tumours) were associated with no difference in the local control but with a decreased radiation resistance for hypoxic tumours shown by a better local control at 4 years compared with photons [52]. In locally advanced pancreatic cancers, which are very hypoxic tumours, C-ions combined with gemcitabine improved the 2-year overall survival rates of patients, suggestive of a decreased OER [53]. Relying on Monte Carlo simulations in water, it was demonstrated that, even under hypoxic conditions, C-ions substantially increased the oxygen concentration in the particle tracks, leading to localised water radiolysis and oxidative damage, sufficient to decrease the OER, whereas LET increases [13, 42, 54]. Furthermore, supporting the lack of oxygen effect after C-ions, the same rejoining kinetics of DSBs were reported in oxic and hypoxic conditions after exposure. This is in favour of the same DSBs produced in both conditions but with modification of the contributions of the DNA repair mechanisms over time [55, 56]. However, compared with photons, the repair time was longer after C-ions with more unrepaired damage, and whatever the oxygen concentration, these results support the ‘bomber’ effect of C-ions.

#### Dose fractionation and dose rate

Over the last decade, conventional radiotherapy was improved thanks to the emergence of intensity-modulated techniques and new protocols relying on altered fractionation [57]. In conventional radiotherapy, hypofractionation contributes inducing late effects in normal tissue but also increases tumour response through the reoxygenation of the tumour cells [58]. The physical dose distribution of C-ions allows them to be used hypofractionated. Experiments performed in vitro but also on animals led to the assumption that the efficacy of C-ions and high-LET particles would increase with the dose and the dose per fraction [59, 60]. Moreover, as discussed before, hypoxia reduces local control in solid tumours, and after C-ion exposure, reoxygenation in tumours occurs earlier than with photons, supporting the benefit of short-term fractionated irradiation with C-ions and their ‘bomber’ effect [61].

Besides, during the delivery of C-ions by an active scanning system, there is an important variation in the dose rate corresponding to the irradiation of the deepest parts of the tumour in less than one second and the shallowest parts in a few minutes. Compared with photons, it was shown in HNSCC cell lines that the change in the dose rate from 0.5 to 10 Gy/min does not impact cell survival with C-ions, as well as the residual DSBs [62]. With the emergence of FLASH-Therapy (high dose of ionising radiation delivered at a very high dose rate), which spares normal tissue while preserving anti-tumour activity, experiments with heavy ions at a high dose rate should be performed. Indeed, based on the physical properties of C-ions, the FLASH dose rate could induce an early generation of oxygen extended over the entire tumour, including hypoxic regions, suggesting the high probability of improved efficacy of C-ions [63].

## ‘Stealth’ effect

In parallel with the ‘bomber’ effect, we proposed the concept of the ‘stealth’ effect to explain the superiority of C-ions compared with photons. In this paradigm, because ROS are not generated outside of the path of the radical tracks, the thresholds necessary to trigger the activation of the survival and cell defence mechanisms are not reached, leading to deceiving the cellular defences of the cancer cells by C-ions.

### Lower activation for DNA repair

The DNA repair response is very efficient for simple damage, such as single-strand break or base damage, whereas repairing clustered lesions is highly complex (different types of lesions, numbers of lesions in a cluster or their spatial distribution) and can lead, when inaccurate or delayed, to genetic instability and cell death [64, 65]. Therefore, it is very difficult for any given radiation-induced clustered DNA damage to study the exact mechanism involved and the respective proportion of the two main double-strand break repair pathways, i.e. the canonical Non-Homologous End Joining (NHEJ-c) and the Homologous Recombination (HR) pathways, but also of an alternative pathway such as Microhomology End Joining (MMEJ). Assuming that the main DNA repair pathway activated following photons and C-ions exposure is NHEJ-c, there is some evidence implicating increased usage of the HR after C-ion exposure, mainly due to the generation of short DNA fragments [7, 48, 66]. It could also depend on the cell type for in vitro experiments as well as on the dose, which adds another level of complexity to the understanding of the repair processes [20, 67]. Recently, we and others showed delayed DSB repair and slower kinetics of detection and signalling after C-ions compared with exposure to photons [48, 68]. The exertion of DSB repair detection, assessed through the phosphorylation of ataxia-telangiectacia mutated protein (ATM), was reduced with C-ions, demonstrated by a decrease in the induction of the peak compared with photons. This agrees with the report by Maalouf et al., which underlined that the formation of monomers of ATM in the cytoplasm is required for ATM nucleoshuttling and the following DSB recognition, and is dependent on the LET and radiation type. Furthermore, the phosphorylated histone γH2AX is a DSB marker that allows the monitoring of the DSB resolution [69]. It was shown that ROS production induces H2AX phosphorylation, [70], but also the recruitment of Rad50 and 53BP1. Furthermore, the induction of the peak of γH2AX foci and its decay rate were decreased after C-ions in different populations of cancer cells and their subpopulation of cancer stem cells (CSCs) [48]. Altogether, although linked to the complexity of the clustered lesions, the close and complex relationship between ROS production and the phosphorylation of H2AX may explain the inability of DNA repair pathways to resolve the LMDS after C-ions through a lower detection and signalling of the DSBs. The absence of ROS outside the tracks may contribute explaining these lower detection and signalling, supporting the ‘stealth’ effect of the C-ions.

### No/less activation of the survival and proliferative pathways

**Fig. 4:**
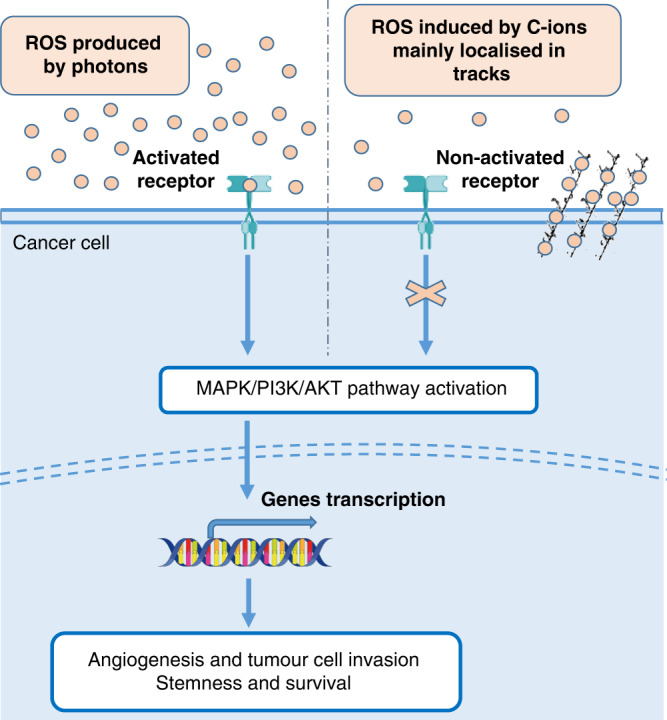
‘Stealth’ effect of C-ions. Because ROS are weakly generated outside the path of the radical tracks induced by C-ions, the thresholds necessary to trigger the activation of the survival and cell defence mechanisms are not reached, leading to deceiving the cellular defences of the cancer cells by C-ions.

If the high efficacy of C-ions is mainly due to the complex and clustered DNA lesions produced, the non-activation of the pro-survival and proliferative pathways after heavy ions exposure may also contribute to explaining their higher RBE. Many actors, such as proteins and mRNA involved in survival and angiogenesis, have been shown to be upregulated after photons and downregulated after C-ions [71] (Fig. 4). Compared with photons, some studies performed in HNSCC cell lines, glioblastoma or lung cancer cells, highlighted the lack of HIF-1α stabilisation in response to C-ions, associated with no or less activation of the pathways involved in radioresistance and angiogenesis [3, 49, 72].

One of the most important pathways involved in cell survival, proliferation, differentiation and radioresistance is the epidermal growth factor receptor (EGFR) pathway. It was reported that activation of EGFR in response to photons, triggered downstream pathways involved in migration, angiogenesis and proliferation [73, 74]. Furthermore, the phosphatidyl-inositol 3-kinase/mammalian target of the rapamycin (PI3K/AKT/mTOR) pathway is involved in survival, cell growth, cancer progression, tumour radio- and chemoresistance, as well as invasion and migration [75]. The activation of EGFR by photons induces the phosphorylation of PI3K, followed by activation of AKT, leading to decreased apoptosis and autophagy associated with enhanced DNA repair recruitment and epithelial-to-mesenchymal transition (EMT) [73]. Besides activation by EGFR, the PI3K/AKT/mTOR pathway can also become activated by photon radiation directly [73]. In contrast to photons, and supporting the superiority of C-ions, no activation of EGFR and downstream pathways was observed with C-ions [76].

In fact, it was shown that C-ions downregulate the AKT/mTOR pathway, thereby inducing autophagy and suppressing cell growth in breast cancer, HeLa or HNSCC cell lines [4, 75, 77]. The phosphorylation of AKT, upstream of mTOR and P70S6K, predicts the radiation resistance of tumours by regulating the repair of DSBs [78]. The phosphorylation of mTOR promotes cell growth and cell cycle progression, and regulates glucose and lipid metabolisms, whereas the phosphorylation of P70S6K kinase induces cell proliferation and survival [75].

Taken altogether, these results show that the biological superiority of C-ions also results from a non-activation of the pro-survival and proliferative pathways as well as from an inhibition of the anti-apoptotic pathways. Because the ROS activate the AKT/mTOR and the Bcl-2/Bax pathways, the studies presented above support the claim that differential activation of the pathways described according to the type of radiation may be attributed to the spatial ROS distribution [79, 80].

### Decrease in the invasion and migration processes

The metastatic potential of cancer cells contributes to the relative failure of conventional radiotherapy [4, 71]. Unlike photons, C-ions do not trigger the migration and invasion processes in cancer cells [5]. Moreover, C-ion irradiation, which is mainly hypofractionated in preclinical and clinical studies, was associated with a decrease in metastasis [81]. Interestingly, some genes involved in the motility and upregulated in response to photons, are downregulated in response to C-ions, thus supporting their potential to suppress the metastatic process [5, 82] (Fig. 4). At molecular levels, the transcriptional factor hypoxia-inducible factor (HIF-1), a masterpiece of the regulation of the response to hypoxia, is involved in the EMT, and its expression is associated with a poor prognosis [72]. Some studies performed in various cell lines, but also in a xenograft model of human non-small cell lung cancer, highlighted no or decreased stabilisation of HIF-1α levels after C-ions with decreased invasion/migration, whereas increased levels of HIF-1α were correlated with increased migration in response to photons [3, 4, 49, 72]. The upstream activation of HIF-1α is mediated by ROS production and particularly OH^•^, suggesting that the concentration of ROS needs to reach a threshold to activate those signalling pathways [3, 4]. In the same way, the MMP-2, a matrix-metalloprotease, which can degrade the connective tissue, and the downstream signalling pathways involved in the EMT (MEK/p38/JNK, AKT/mTOR and STAT3) were activated after photons and decreased after C-ions, in correlation with low migration and invasion capacities [4, 72]. In response to photons, the widespread distribution of ROS in the cells can activate HIF-1α and the EMT pathways, whereas the ROS might not be sufficient outside the tracks to stabilise HIF-1α and the upstream EMT-activated kinase cascades. These results support the importance of the spatial ROS distribution at the nanometric scale to deceive the initiation of the invasion/migration processes, and thus the ‘stealth’ effect of C-ions.

### Stemness and survival

Cancer stem cells are major contributors to the resistance to photons because they present self-renewal and invasion capacities, but also resistance to chemotherapy and conventional radiotherapy due to enhanced DNA repair abilities and reduced ROS levels [5, 48, 83, 84]. However, many data suggest that C-ions kill CSCs more efficiently than photons, both in vitro and in vivo [3, 5, 83]. Several processes may explain these biological advantages of C-ions and are rather in favour of the ‘bomber’ effect, such as the previously described decrease of OER or their efficacy independent of the *p53* status. However, some arguments are totally in favour of the ‘stealth’ effect in order to explain that C-ions overcome the resistance to treatments due to the presence of CSCs. The CSCs can acquire radioresistance through the activation of the anti-apoptotic protein Bcl-2 and the survival AKT/mTOR pathway [85]. Therefore, we speculate that C-ions, which depress the AKT survival pathway, may target the CSCs more efficiently [2, 86]. Furthermore, it was shown that ROS production activates the PI3K/AKT/mTOR pathway through the transforming growth factor-β (TGF-β) upregulation but is also involved in the activation of many transcription factors, such as Snail, zinc finger E-box binding homeobox 1 (ZEB1), signal transducer and activator of transcription 3 (STAT3), Wnt, Hedgehog or Notch [87]. Some of these pathways greatly contribute to the maintenance of CSCs and, therefore, to their radioresistance [2]. Although CSCs display enhanced protection from oxidative damage, many genes involved in their maintenance, self-renewal, migration and invasion abilities or pro-survival pathways are activated and modulated by ROS production. Consequently, we suggest that the differential ROS distribution after photons and C-ions could explain the higher efficacy of C-ions on CSCs by deceiving the activation of these pathways through a ‘stealth’ effect.

## At the frontier of the ‘stealth-bomber’, a grey zone

### ‘Non-targeted’ effects

Recently, increasing interest in the ‘non-targeted’ effects of the C-ions has been highlighted, among which is the bystander effect. Up to now, little data were available with heavy ions and therefore, questions remain unanswered whether the non-targeted effects or the intercellular effects are similar in response to photons or C-ions. Radiation-induced bystander effects (RIBEs) could occur in cells not traversed by C-ions by the exchange of molecules through gap junctions or by the release of cytokines [88]. These secreted factors or connected channels could induce the activation of the immune system, as well as the suppressing effects [2]. Currently, RIBEs were identified to be induced by ROS, reactive nitrogen species (RNS) or cytokines such as TGF-β which can activate the MAPK pathways and the nuclear factor-kB or the release of interleukine-8 [2, 88, 89]. RIBEs are responsible for DNA damage, chromosome aberrations or cell death [90]. Besides cytokines, extracellular vesicles or exosomes containing factors such as proteins or mRNA have been described as responsible for RIBEs (DNA damage or activation of signal transduction) on the neighbouring cancer cells [88]. In response to photons, there is no doubt about the involvement of the exosomes in RIBEs. Their role in response to heavy ions needs to be further studied [91, 92].

### Immune response

Numerous clinical trials are ongoing because the combination of photons with immune checkpoint inhibitors is efficient. However, more studies are required to support the benefits of this combination with C-ions, which requires an understanding of the exact immune mechanisms involved. Although many studies established the recruitment of immune cells close to the tumour site in response to photons, only a few studies showed anti-tumour immunity and a trend towards higher cytokine release by the tumour cells after C-ions [93]. An extension to different LET and models is needed. Recently, studies performed on cell lines irradiated with photons deciphered molecular pathways associated with DNA damage and involved in the radiation-induced immune response, such as three prime repair exonuclease 1 (TREX1)-Exosome, Stimulator Of Interferon Response CGAMP Interactor (STING)-type I interferon, and STING-independent ATR-Chk-IRF axes [94, 95]. A recent study showed a dynamic change in the gene expression of an oesophageal cancer cell line after photons and C-ions from 6 to 24 h after irradiation, before becoming similar over time after a few days [96]. This could be indirectly attributed to different patterns of DNA damage produced according to the type of irradiation, but also to the changes resulting from cell stress. However, the current studies are mostly biased because they do not consider the microenvironment and immune cells, which are key actors in the immune response. After photon exposure, a depletion of Ku70/80 proteins has been shown to enhance Programmed Death-Ligand 1 (PD-L1) expression in cancer cells [95]. Compared with photons, it was also shown that C-ions induced upregulation of PD-L1 expression on the surface of human osteosarcoma cells, whether at both physical or biological equivalent doses [97]. All these data reinforced a promising approach relying on a combination of immune therapy and carbon therapy to target radioresistant tumours. More recently, ROS and RNS were shown as key effectors of innate immunity and activators of some immune pathways [98]. The dose distribution, and consequently the spatial ROS distribution, probably constitutes a central parameter in the modulation of the immune response, which needs to be further investigated.

## Conclusion

In this review, we focused on the discussion of the biological effects of C-ions, which can be attributed to the clustered spatial ROS distribution at the nanometric scale and could explain the higher efficacy of C-ions compared with photons. We have presented evidence that when biological targets are on the ionising tracks of the particles, the biological advantages of C-ions can be compared with a ‘bomber’ effect. However, when biological targets are outside the track, the very low production of ROS out of the tracks does not reach the threshold necessary to trigger pathways such as pro-survival or invasion/migration pathways, and, therefore, deceives the cellular defence of the cells, producing a ‘stealth‘ effect (Fig. 2). Although the ‘bomber’ effect of C-ions has been extensively described, the ‘stealth’ effect has not. The evidence of the ‘bomber’ effect arises from the capacity of C-ions to produce complex and clustered DNA lesions, as well as isochromatid breaks reflecting the track structure of the heavy ions. The scientific community still debates the proportion of the relative contribution of direct and indirect effects. The same observation could be made for the ‘stealth-bomber’ paradigm. The ‘stealth’ effects, if more difficult to quantify than the ‘bomber’ effects, cannot be neglected and are probably underestimated. They certainly play a central role in the biological efficacy of carbon ions on the tumour, combined with fewer adverse effects.

The non-targeted effects in relation to the immune response after C-ion exposure seem also to support the ‘stealth-bomber’ effects and need to be further investigated. Furthermore, the paradigm of the ‘stealth-bomber’ effect of C-ions needs to be validated with protons and higher-LET particles. The ‘bomber’ effect is predicted by NanOx, a multiscale model that integrates the chemical aspects of the interaction of radiation and matter, and takes into account the stochastic nature of radiation at the nanometric and micrometric scales [99, 100]. Further perspectives of the NanOx model will be to predict the ‘stealth’ effects. Because spatial ROS distribution is the masterpiece of this theory, the combination of ultra-high dose rate of irradiation (FLASH), which generates high concentrations of ROS, and high-LET could constitute a promising therapeutic strategy supporting the ‘stealth-bomber’ effect of C-ions. Finally, our ‘stealth-bomber’ paradigm supports the higher biological efficacy of C-ions compared with photons and highlights the need to expand their clinical use.

## Data Availability

This review was designed and written according to the literature available on PubMed.
